# Nonclonal coloniality: Genetically chimeric colonies through fusion of sexually produced polyps in the hydrozoan *Ectopleura larynx*

**DOI:** 10.1002/evl3.68

**Published:** 2018-07-11

**Authors:** E. Sally Chang, Maria E. Orive, Paulyn Cartwright

**Affiliations:** ^1^ Department of Ecology and Evolutionary Biology University of Kansas Lawrence Kansas 66045

**Keywords:** Coloniality, chimerism, evolutionary genomics, genetic variation, hydrozoa, genetic conflict, life‐history evolution, RAD‐seq

## Abstract

Hydrozoans typically develop colonies through asexual budding of polyps. Although colonies of *Ectopleura* are similar to other hydrozoans in that they consist of multiple polyps physically connected through continuous epithelia and shared gastrovascular cavity, *Ectopleura larynx* does not asexually bud polyps indeterminately. Instead, after an initial phase of limited budding in a young colony, *E. larynx* achieves its large colony size through the aggregation and fusion of sexually (nonclonally) produced polyps. The apparent chimerism within a physiologically integrated colony presents a potential source of conflict between distinct genetic lineages, which may vary in their ability to access the germline. To determine the extent to which the potential for genetic conflict exists, we characterized the types of genetic relationships between polyps within colonies, using a RAD‐Seq approach. Our results indicate that *E. larynx* colonies are indeed comprised of polyps that are clones and sexually reproduced siblings and offspring, consistent with their life history. In addition, we found that colonies also contain polyps that are genetically unrelated, and that estimates of genome‐wide relatedness suggests a potential for conflict within a colony. Taken together, our data suggest that there are distinct categories of relationships in colonies of *E. larynx*, likely achieved through a range of processes including budding, regeneration, and fusion of progeny and unrelated polyps, with the possibility for a genetic conflict resolution mechanism. Together these processes contribute to the reevolution of the ecologically important trait of coloniality in *E. larynx*.

**Impact Summary**

Coloniality is a life‐history trait that confers great competitive advantage for space in crowded benthic environments. The method by which hydrozoans (phylum Cnidaria) create large colonies to compete for space is through asexual budding of genetically identical individuals that remain attached to one another through continuous epithelia and shared gastrovascular cavity. The colonial hydrozoan *Ectopleura larynx* however cannot bud indeterminately and instead achieves its large size by fusing sexually produced juvenile polyps to the adult colony.

This process appears contrary to what is predicted by evolutionary theory—genetically chimeric colonies give rise to potential genetic conflict between colony members sharing resources, and thus might be opposed by natural selection. To determine the degree of genetic diversity within a colony and whether or not there is a genetic homogenization process, such as somatic cell parasitism, we used a genomic approach to assess genetic variation within and between colonies. We found that *E. larynx* colonies represent chimeric collections of individuals, including distantly related polyps. Estimates of genome‐wide levels of relatedness for polyps within and between colonies confirm the potential for genetic conflict, despite a background of low genetic diversity in this system. Our results provide an evaluation of genomic diversity in a system that uniquely uncouples coloniality from strict clonality, and suggests that in certain situations selection for large size takes precedence over selection for genetic homogeneity within an integrated colony, producing colonies containing a complex set of genetic relationships.

Coloniality is a key evolutionary innovation that confers a strong advantage over solitary organisms in substrate‐limited marine environments by allowing for rapid colonization and spread over available substratum (Jackson 1977; Coates 1985). Amongst most hydrozoans (phylum Cnidaria) coloniality is achieved through asexual budding of polyps that remain physically attached by continuous epithelia and a shared gastrovascular cavity. Recent findings reported that the hydrozoan *Ectopleura larynx* Ellis and Solander, 1786 does not asexually bud polyps indeterminately, but rather achieves large colony size through the aggregation and fusion of sexually produced offspring (Nawrocki and Cartwright 2012) (Fig. 1). Not only do the sexually produced offspring settle upon established colonies, Nawrocki and Cartwright (2012) demonstrated that the epithelia of the polyp and the colony eventually become fused such that the gastrovascular cavity is shared throughout the entire colony. The end result is indistinguishable from other hydrozoans that achieve this level of integration through asexual budding. The formation of colonies through an amalgamation of sexually reproduced polyps in *E. larynx* has important evolutionary and genetic consequences. If individual colonies of *Ectopleura larynx* are mixtures of genotypes, then a potential source of conflict exists between distinct genetic lineages, which may vary in their ability to access the germline and achieve representation in the gametes produced by the colony. The most extreme form of this conflict is germline parasitism where one lineage monopolizes the reproductive output while contributing only partially to the somatic functioning of the colony (Buss 1982). Experimental evidence of successful germline parasitism by a particular lineage has been noted in the colonial tunicate *Botryllus schlosseri* (Stoner and Weissman 1996; Stoner et al. 1999) and in the social slime mold *Dictyostelium* (Buss 1982; Noce and Takeuchi 1985; Ennis et al. 2000).

**Figure 1:**
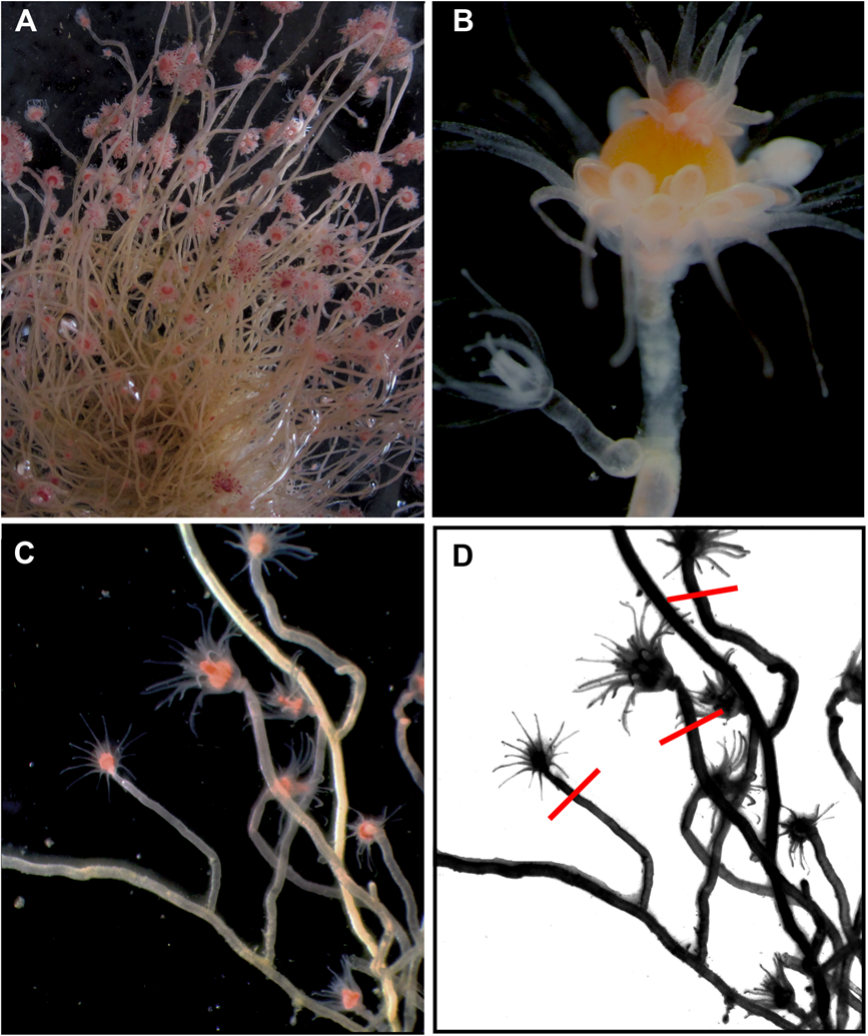
Ectopleura larynx. (A) *E. larynx* colony. (B) Juvenile polyp after recent settlement on adult, female colony. (C) Juvenile polyps sharing continuous tissue with rest of adult colony after complete fusion. (D) Schematic version of Panel C, with red lines indicating approximate cut sites for polyp harvesting.

Alternatively, the germline of a colony formed by fusion of polyps may display germline chimerism in which multiple germline lineages persist and potentially produce gametes, leading to conflict over allocation of reproductive resources to gametes formed from different genotypes. There is evidence of the existence of multiple genotypes in gonad tissue after the fusion of two colonies in *Botryllus schlosseri* (Pancer et al. 1995; Carpenter et al. 2011) and of the long‐term persistence of chimerism in colonies of some corals (Puill‐Stephan et al. 2009). Given that *E. larynx* colonies are formed by a process that includes polyp fusion, either of the above scenarios of conflict may exist within an *E. larynx* colony.

There are many examples of selection for mechanisms to keep colonies genetically homogenous to prevent competition between genetic lineages (i.e., to not allow multiple genotypes to produce gametes), especially for benthic, sessile organisms where different colonies may unavoidably come in contact with one another and need to prevent fusion (Buss 1987). Indeed, genetically encoded allorecognition systems, which only allow colony fusion within certain bounds of relatedness, are well‐defined in the hydrozoan *Hydractinia* and in *B. schlosseri* (reviewed in Rosengarten and Nicotra 2011). Likewise, there is theoretical evidence for the effectiveness of within‐organism selection between cell lineages and forms of somatic growth in decreasing within‐colony genetic differentiation in the face of somatic mutations (Otto and Orive 1995; Otto and Hastings 1998; Orive 2001). Recent empirical work in both plants and animals has demonstrated mechanisms for both an increased opportunity for within‐organism selection (Burian et al. 2016) and a reduction in the opportunity for between‐genotype competition for reproduction (Barfield et al. 2016). In coalescing red algae, which exhibit high levels of genetic chimerism from fusion (González and Santelices 2017), differences between growth rates between lineages of cells may serve to segregate the lineages into different axes, thus reversing chimerism in upright axes or branches after fusion as one cell lineage outcompetes the others (Santelices et al. 2016).

In *E. larynx*, whose ancestors were solitary and lost the ability to form extensive colonies through asexual budding (Cartwright and Nawrocki 2010; Nawrocki and Cartwright 2012), the fierce competition for space on marine surfaces and other size‐related mortality factors (Jackson 1977; Coates 1985) may have driven the reevolution of colonies that are a compromise as far as genetic homogeneity. Colonies of *E. larynx* may represent an evolutionary “kluge” of sorts where the pressure to form large colonies takes precedence over preventing germline–soma or germline–germline competition.

*Ectopleura larynx* belongs to the hydrozoan clade Aplanulata (Collins et al. 2005; Nawrocki et al. 2013). Most members of Aplanulata lack a free‐living planula larvae stage and instead brood offspring inside the gonophores of the mother, until the juvenile polyp (actinulae) stage. Within Aplanulata, coloniality has been lost and most members of this clade, including the model organism *Hydra*, are solitary. In the lineage leading to *Ectopleura*, the ability to continually bud asexually in an adult colony, leading to indeterminate growth, was also lost, although they display a remarkable ability to regenerate tissues when injured (Nawrocki and Cartwright 2012; Nawrocki et al. 2013).

It is within this evolutionary context that *Ectopleura* reevolved coloniality via polyp fusion. This is the only known instance of reevolution of a fully integrated colonial phenotype in the Hydrozoa (Cartwright and Nawrocki 2010), with different species within *Ectopleura* displaying varying levels of coloniality. In *Ectopleura larynx*, male colonies release sperm and fertilize local female colonies which then brood their offspring. If a new polyp lands on available substratum, as opposed to an established colony, it will branch 4–6 polyps off its apical end to establish the new colony in an initial phase of determinate growth, but will not proceed beyond this size using the asexual budding process (Petersen 1990; Schuchert 2006; Nawrocki and Cartwright 2012). New polyps can also form as the result of an apparent tissue‐damage response when the colony is preyed upon by nudibranchs (P. Cartwright, pers. obs.). Apart from during early development and in the regenerative response to tissue damage, *E. larynx* colonies have never been observed to spontaneously asexually bud polyps (Nawrocki and Cartwright 2012). Instead it appears that *E. larynx* colonies achieve their large size (dozens to hundreds of polyps per colony) through the aforementioned fusion of sexually produced juvenile polyps from the local population of colonies, ultimately producing colonies that may contain multiple genetic lineages.

Here, we characterize the extent to which these different processes (initial budding, regeneration, fusion, and any potential genetic homogenization/somatic takeover mechanism) contribute to the formation of *E. larynx* colonies. Using a RAD‐seq approach, we determined the genetic diversity within and between colonies and characterized the types of genetic relationships (i.e., familial, clonal) present within colonies of *E. larynx*. Further, to estimate the potential for genetic conflict, we made genome‐scale estimates of relatedness to serve as a proxy for the probability of matching or mismatching at a genetic conflict locus. This represents one of the first genome‐scale studies of diversity within single colonies of an animal. This unique system, which potentially decouples the effects of coloniality and strict asexual reproduction, will allow us to investigate the effects of coloniality and clonality on the evolution and genetics of a species, as well as the interplay between selection for spatial competition and selection for genetically homogenous colonies.

## Materials and Methods

### SAMPLING OF ECTOPLEURA LARYNX

Specimens of *Ectopleura larynx* were collected from eight locations along the coasts of Maine and Northern Ireland. Colonies were removed from the edges of docks or from submerged rocks in shaded subtidal areas. Sex was determined morphologically for each colony and 5–15 polyps per colony were chosen at random from colonies composed of approximately 25–100 polyps (Fig. 1C). The polyps (Fig. 1D) were stored individually in ethanol for DNA isolation.

### DNA EXTRACTION/LIBRARY CONSTRUCTION/SEQUENCING

DNA was extracted from each polyp using the QIAGEN DNeasy Blood and Tissue kit. To obtain genome‐wide sequencing representing loci across the entire genome of *E. larynx*, we took a restriction site‐associated‐digest (RAD‐Seq) approach (Miller et al. 2007; Baird et al. 2008) using a modified multiplex‐shotgun‐genotyping method (Andolfatto et al. 2011) as implemented in Monnahan et al. (2015), with modifications and barcoding of samples as outlined in Supplemental Methods. One hundred ninety‐two samples were prepared in two 96‐sample batches for sequencing, with assistance from the University of Kansas Genome Sequencing Core (GSC; Lawrence, KS). Illumina sequencing of the two libraries was performed by the GSC in one lane each of high‐output paired‐end 100bp reads on an Illumina HiSeq 2500 System, although only forward reads were used in further analyses.

### READ QUALITY FILTERING/RECONSTRUCTION OF POLYMORPHIC LOCI USING STACKS

After sequencing, the raw Illumina sequence data was quality‐filtered and demultiplexed into sample‐specific FASTQ files using the process_radtags program of Stacks v.1.44 (Catchen et al. 2013) on default settings. A series of consistently low‐quality bases had to be omitted from the middle of reads from one of the two libraries, so the same 14bp were trimmed from the second library as well, leaving 86bp reads for further analysis. At this stage, sequences for several polyps that had fewer than <10,000 retained reads after the process_radtags step were removed from further analysis.

We utilized the Stacks pipeline (Catchen et al. 2013) for *de novo* assembly of restriction‐site‐associated loci and identification of SNPs for further analysis. The pipeline was run twice for the purposes of comparison of major results: Once with entirely default parameters for each program except those enabling parallel execution, and once with additional adjustments made in the UStacks module to increase the minimum stack depth required to build a locus (‐m 6) and to enable the resolution of overmerged tags (‐d). These adjustments were chosen to prevent both loss of alleles due to insufficient read depth, and false merging of multiple loci into a single stack.

### COLONY‐LEVEL AND POPULATION‐LEVEL FILTERING OF INDIVIDUALS AND POLYMORPHIC LOCI

For investigation of within‐colony relationships without any effects of missing data, we retained for each colony through the Stacks “populations” module only those polymorphic sites present in all polyps in a colony (r 1.0) and with at least eight reads per site/per individual (‐m 8). For within‐colony investigations of relationships and genetic diversity, only colonies that contained more than four polyps that successfully passed previous filtering steps and had at least 100 associated SNPs were retained, resulting in a dataset containing 19 colonies from two populations in Maine and two in Northern Ireland (Tables S1 and S2). Details about the datasets used for population‐level calculations are available in Table S3 and Supplemental Methods.

### DETERMINATION OF WITHIN‐COLONY RELATIONSHIPS USING GENETIC DISTANCES

**Figure 2:**
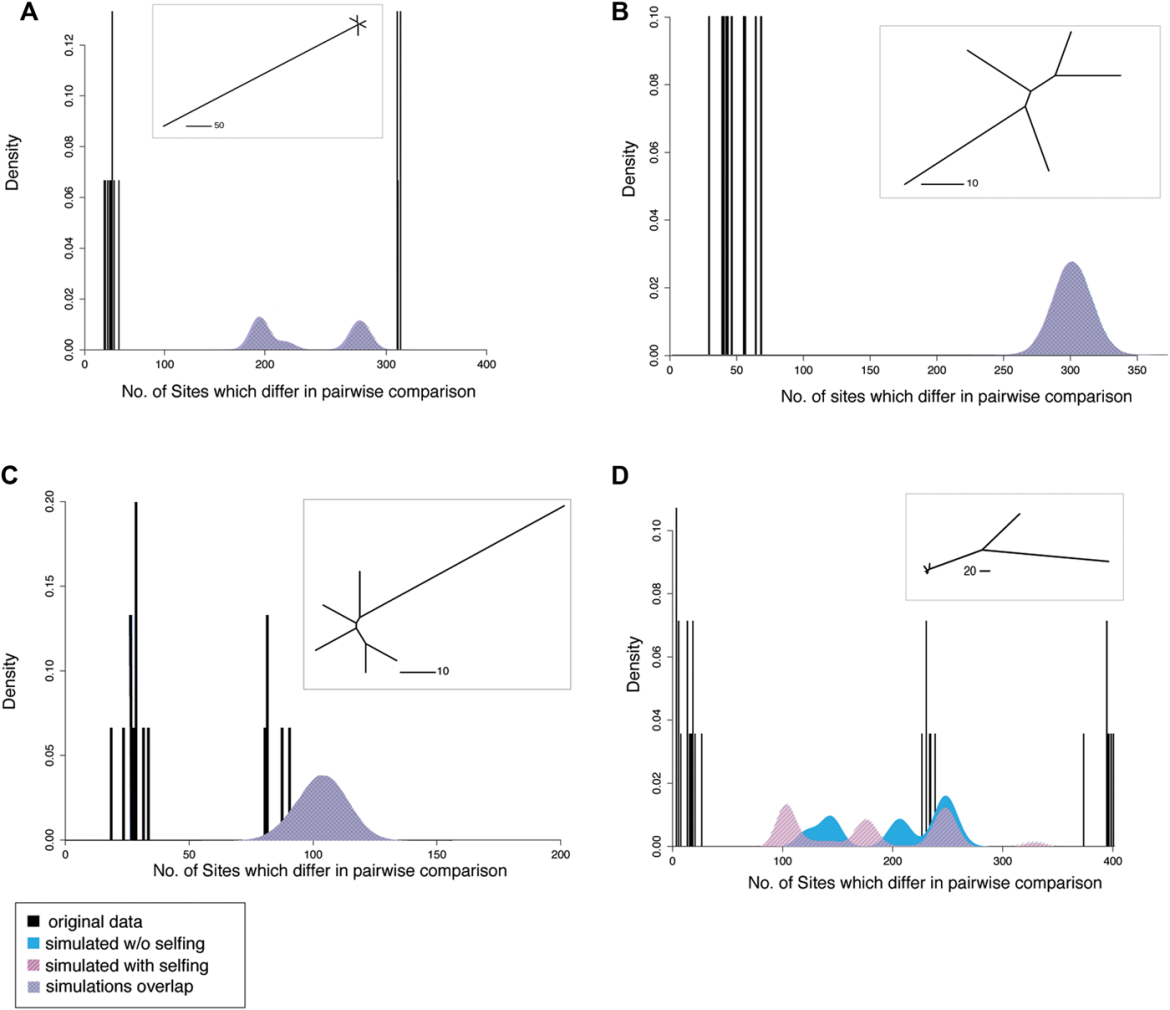
Genetic relationships within selected colonies of *E. larynx*. Actual genetic distances (black bars, calculated as the number of loci which differ in each pairwise comparison) between polyps of *E. larynx* in every possible pairwise comparison in a colony, and those predicted under simulations of sex with selfing (pink), without (blue) and where those simulated distributions overlap (violet), for colony ME2.2 (A), ME1.3 (B), ME1.7 (C), and ME2.3 (D). Insets are neighbor‐joining trees with each branch representing a polyp within the colony, branch lengths representing number of differing polymorphic sites.

A major goal of this study was to determine whether polyps within a colony are part of the same genetic lineage (i.e., matching multilocus lineages with differences likely only due to sequencing error or somatic mutation) or if there were more distant types of relationships present within the colony resulting from fusion, which, to our knowledge, has never been assessed at a genome‐scale for a hydrozoan colony of any species. To do so, we compared genetic distances to those expected under sexual reproduction within a given colony of *E. larynx* both with and without selfing using the R package RClone (Bailleul et al. 2016) (further information in Supplemental Methods). In our case, because colonies are a single sex, and therefore within‐colony reproduction is not actually possible, the results of these simulations represent a hypothetical distribution of genetic distances to which we can compare the actual observed within‐colony distances, allowing for identification of putative clones. Although the selfing simulations do not represent a biologically feasible mode of reproduction given single‐sex colonies, they served as an important visual contrast for the lowest limits of possible nonclonal diversity. We categorized the relationships between polyps in a given colony into three overall categories based on the relation of their genetic distances to those simulated as products of reproduction within a colony: (Type I) comparisons that produced distances less than expected due to sexual reproduction; (Type II) comparisons with distances within the range expected due to sexual reproduction; and (Type III) comparisons corresponding to genetic distances greater than expected due to theoretical within‐colony sexual reproduction. Per‐colony results of these simulations are presented in Figure 2 and Figure S1.

### EVALUATING PATTERNS OF INTRACOLONIAL ALLELIC SEGREGATION

To further understand the types of relationships present within a colony, we examined patterns of allelic segregation between polyps at all SNPs in the given colony's dataset using custom R scripts. The three genetic relationship categories we discriminated between were: (A) genotypes whose only genetic differences were ones that could be explained solely by sequencing error or somatic mutation within a clonal genotype; (B) genotypes that can be explained by Mendelian segregation of alleles found within the main clonal lineage; and (C) genotypes containing alleles not found in other polyps in the colony, giving possible evidence of gene flow from outside the main clonal lineage. We further divided this last category into whether or not genotypes differed by one or both alleles from the genotypes of other polyps in the colony. Mismatching at both alleles as compared to other polyps was taken as evidence of fusion from unrelated polyps. A detailed description of this approach is presented in Supplemental Methods.

**Figure 3:**
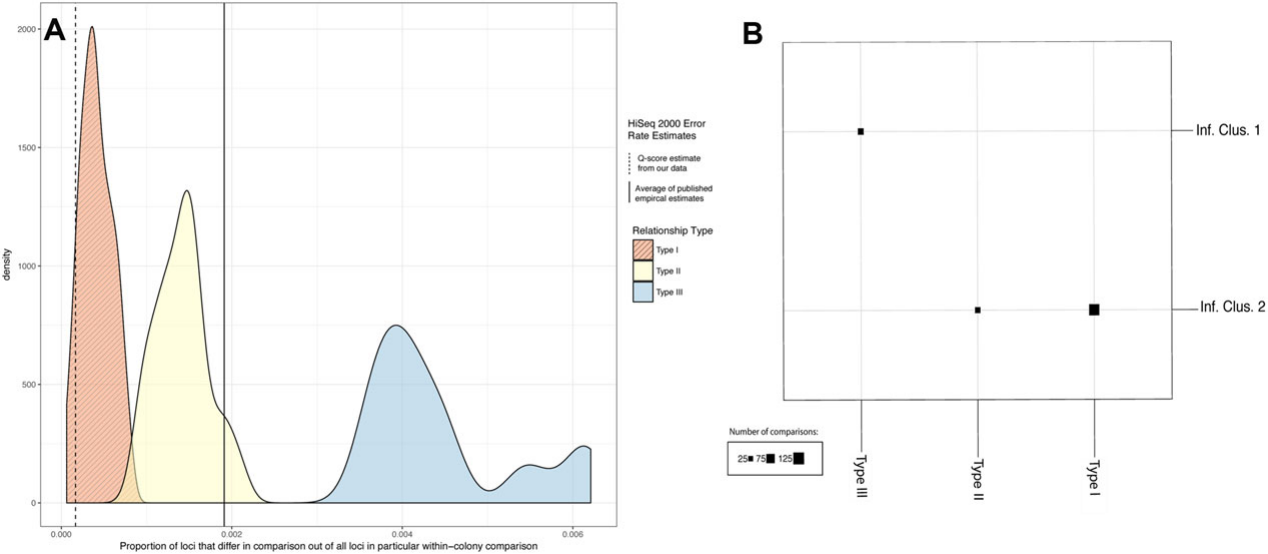
Density distributions of the combined pairwise genetic distances for each of three classes of Relationships (see Text for details), and results of clustering these pairwise comparisons via k‐means clustering, as compared to relationship types proposed via simulation approach. Panel (A) Q‐score estimate is the probability that a given base call is incorrect calculated from the average posttrimming Phred score for entire dataset (37.79). The average published estimate is the average (.00191, SD = .00027) of three independent empirical studies quantifying substitution error rates on the HiSeq 2000/2500 line of sequencers (Minoche et al. 2011; Wall et al. 2014; Schirmer et al. 2016). Panel (B) depicts membership in two inferred clusters (rows), as compared with membership in the three types of relationships inferred via the simulation approach (columns). Type III relationships (those that are larger than expected due to within‐colony sexual reproduction), fall in a distinct, nonoverlapping cluster from Types I and II.

Because our data (proportion of sites that differ in a given comparison) may violate assumptions of an ANOVA (namely those of normality and independence), we conducted k‐means clustering to test for the presence of distinct clusters that matched the three relationship types we described previously (see Supplemental Methods for details). Assessment of optimal number of clusters was carried out in fviz_nclust() function from R package factoextra v.1.05 (Kassambara and Mundt 2017) and the NbClust() function from the NbClust package (Charrad et al. 2014) (Figure S5 and Table S5). Actual k‐means clustering of relationships by genetic distance was carried out using the kmeans() function from base R. Density plots to visualize these distances were created using the ggplot2 R package (Wickham 2009), using the same dataset as for the k‐means clustering process (Fig. 3).

### ASSESSING THE EFFECTS OF READ‐DEPTH AND ALLELIC DROPOUT ON OUR RESULTS

One potential issue with RAD‐sequencing based techniques, as opposed to Sanger‐sequenced genetic markers such as microsatellites, is loss of one allele in a true heterozygote, creating false homozygotes in the resultant dataset. This “allelic dropout” can either be due to low sequencing depth of one of the two alleles, meaning that during the SNP‐calling phase these sites are called as homozygotes, or due to a genuine mutation in one of the two alleles such that it no longer has a restriction‐enzyme cut site (Gautier et al. 2013; Davey et al. 2013). It might be expected that the former issue will be most prevalent when sequencing depth is low, while the latter can be diagnosed by the presence of low read depth due to the loss of the RAD‐sequences associated with the null allele (Gautier et al. 2013). Given this, it was important to assess whether or not read‐depth was roughly the same for all loci, particularly for sites that had the alternate homozygote in comparison to the other polyps in the colony, by comparing distributions of read depth at those sites both graphically and statistically using functions from the vcfR package v1.4 (Knaus and Grünwald 2017) and the t.test() and Wilcox.test() functions from Base R v.3.3.3 (Team 2017).

### GENOMIC ESTIMATES OF RELATEDNESS BETWEEN COLONY MATES

To further understand the potential for either cooperation or conflict between polyps in a given colony of *E. larynx*, we determined whether polyps within a colony are more closely related to one another than polyps from different colonies. Such an analysis will determine whether different alleles at loci involved in conflict would likely interact with one another in a colony, even given the low amount of background genetic diversity observed in these samples. One traditional measure of the potential for such conflict is Hamilton's *r* (relatedness), defined as the probability that a recipient individual carries an allele identical by descent with an allele sampled randomly from a donor (Charlesworth and Charlesworth 2010). For a relevant locus, the higher the relatedness between individuals, the less potential for genetic conflict exists between individuals with respect to this locus.

For large SNP datasets, one proxy for estimating relatedness of two individuals is to estimate the average probability of a match between alleles drawn at random from each individual, under the assumption that sequence identity implies identity by descent (approach reviewed in Speed and Balding 2015). The probability of identity by descent for two alleles sampled from two different individuals (i.e., a kinship coefficient) is directly related to the potential for genetic conflict as it should be half of Hamilton's *r* in the absence of inbreeding. One algorithm that can calculate this probability for SNP data is KING‐robust (Manichaikul et al. 2010). This algorithm has the added benefit of being robust to unknown background population structure, which we have little knowledge of in *E. larynx*. Using the KING‐robust algorithm as implemented in the “‐relatedness2” option of VCFtools v. 0.1.15 (Danecek et al. 2011), we calculated **φ** (kinship coefficient) for all possible pairs of polyps in a collecting location, both within and between colonies.

To further quantify the probability that individuals in a given colony will mismatch or not at potential allorecognition loci in the absence of knowledge of specific loci, we utilized the “—genome” option in the software package PLINK (Purcell et al. 2007) to calculate probabilities of identity by descent at a random SNP for 0 alleles, 1 allele, and both alleles for every possible pair of two individuals.

To create a larger dataset of independent SNPs for estimating relatedness between colonies, we relaxed some SNP filtering and turned on the –write_single_snp option in the Stacks populations module (see Table S3). Although this increased the amount of missing data, it allowed for an increased number of loci to consider genome‐scale, between‐colony estimates of relatedness. Measures of relatedness could be affected by the presence of unknown inbreeding, but we find that there is limited evidence for inbreeding in this system (see estimates of F_IS_ in Results section).

## Results

Sequencing, read filtering, and subsequent loci reconstruction resulted in a dataset containing 19 colonies from four of the sampled locations, two in Ireland and two on the coast of Maine, and hundreds of loci per colony that had no missing data and sufficient depth of coverage (Table S2).

### DETERMINATION OF RELATIONSHIPS VIA SIMULATIONS

We generated and visualized simulated distributions of genetic distances (as number of loci that vary in a pairwise comparison) between offspring resulting from hypothetical within‐colony sexual reproduction, and then plotted our actual within‐colony, between‐polyp pairwise genetic distance data on the same graphs (Fig. 2). Our hypothetical distributions served as reference for the possible amount of genetic variation among offspring that could be produced by within‐colony genetic variation alone. We categorized the relationships between polyps in a given colony into three categories based on the relationship of their genetic distances to those simulated as described in the Methods section.

Some combination of these relationships/distance classes was present in each given colony (Fig. 2, Fig. S1). Notably, 18 out of 19 colonies examined possessed Type I (clonal) comparisons and 16 contained some combination of Type II and Type III. There were no significant positive or negative associations between the possession of Type II and III relationships (*P*‐value for Fisher's exact test of association between two categorical variables is 0.6649). Initial examination of the presence of these distance classes in the sets of loci resulting from our “default” and “conservative” Stacks loci reconstruction runs yielded identical results, so the “default” dataset was not investigated further.

### COMPARISON OF GENETIC DISTANCES

Visually, the three types of within‐colony relationships described above had distinct, nearly nonoverlapping genetic distances (Fig. 3). A majority of methods to select an optimal number of clusters (K) selected only two clusters as the minimal K to explain most variation in the data (Fig. S5 and Table S5), although some measures did select values of K greater than two. K‐means clustering confirmed that grouping the comparison data into only two groups explained >88% of the variation in this data set, although expanding the analysis to three clusters does explain more of the data (93.2% total). Type III relationships (those that are larger than expected from within‐colony sexual reproduction), fall in a distinct, nonoverlapping cluster from Types I and II (Fig. 3B).

The average Phred score for our data set after quality‐filtering of reads was 37.79, corresponding to a Q‐score, or the probability that a particular base is incorrectly called, of just .00017. The Type I and some Type II genetic distances are greater than our Q‐score error estimates and most are less than published empirical estimates (Minoche et al. 2011; Wall et al. 2014; Schirmer et al. 2016) of Illumina HiSeq sequencing error rates from studies using similar sequencing approaches (HiSeq 2000/2500, looking at error rates from R1 (forward) reads, including preanalysis quality filtering). All Type III relationships are greater than both of these estimates (Fig. 3A). Although all of Type I and most of the Type II relationships fall below published estimates of sequencing error, the existence of two distinct peaks for Type I and Type II relationships and our further examination of individual SNPs (below) indicate that Type II comparisons are distinct from the between‐clone comparisons with sequencing error that comprise the Type I comparisons. Taken together, the visual and statistical evidence suggests the presence of at least two, and likely three, distinct classes of relationships among our within‐colony genetic distance data. Type III relationships particularly (those greater than expected according to a simulated model of sexual reproduction), represent a unique class of within‐colony genetic relationships.

### EXAMINATION OF INDIVIDUAL LOCI

For each site that was polymorphic amongst Type I relationships in a colony, we examined whether it appeared to be evidence of single, unique sequencing errors, somatic mutation or other forms of actual divergence between putative clones. We found that 79.3% of all SNPs with the Type I clonal category are found in just a single within‐colony comparison, suggesting that they are random sequencing error. Of sites polymorphic between clones, 19.3% of them contained two repeated allelic configurations that are one mutational/error step apart, suggesting the possibility of colony‐specific somatic mutation. Finally, just 1.3% of sites polymorphic between clones are not fully explicable by either of the processes above (more than two alleles per site or one polyp being separated by more than one mutational/error step from the others).

Next, we examined the loci polymorphic in the other two types of relationships to determine if fusion of polyps from other colonies or from polyps not sampled in this study was responsible for some of the genetic divergence between polyps in these comparisons. In particular, we identified a class of loci where polyps were a different homozygote from the other polyps in the same colony. That is, the divergence in these sites could not be explained as solely as products of Mendelian segregation of the clonal alleles or a single step of somatic mutation or sequencing error from other genotypes in the colony. Given the extremely low probability of somatic mutation occurring twice at a given site (Orive 2001), the existence of these distinct homozygotes can be taken as evidence of the presence of multiple distinct genotypes in a given colony that are not consistent with mosaicism through random somatic mutations, and so are likely the products of chimeric fusion (Schweinsberg et al. 2015; Schweinsberg et al. 2016; Schweinsberg et al. 2017). All Type III comparisons and some Type II comparisons possessed sites that differed in this manner.

To rule out the possibility that this pattern of divergence was generated by dropout of one allele in either genotype in a comparison (i.e., either polyp could be a false homozygote) due to low sequencing depth, we compared the distributions of per‐colony read depth between sites a which this pattern occurred versus all other sites. We found that the shape and location of the distributions of read depths were nearly identical (Fig. S2) and did not have a significantly different mean (*t* = 1.6338, df = 4748.8, *P*‐value = 0.1024) or overall location/shape (Mann–Whitney U Test, = 0, W = 56071000, *P*‐value = 0.6064).

### POPULATION‐LEVEL GENETIC DIVERSITY

At each collecting location, the distributions of between‐colony and within‐colony genetic distances are largely overlapping, indicating that there are some within‐colony comparisons that are just as large as between‐colony comparisons, and that there are some extremely similar genotypes present in multiple distinct colonies (Fig. S3). The presence of these nearly identical genotypes in different colonies may represent fusion of polyps produced through matings between close relatives or may also simply be an artifact of having few polymorphic loci with which to distinguish individuals.

Calculations of relevant diversity parameters considering all sites, both variant and invariant, are presented in Table 1. Overall, populations of *E. larynx* display low levels of polymorphism and allelic diversity (**π)** when compared to other location‐level, RADseq‐based estimates of genomic diversity of marine invertebrates, even considering other clonal and/or colonial cnidarians (Reitzel et al. 2013; Bellis et al. 2016; Drury et al. 2016; Drury et al. 2017; Gleason and Burton 2016; Xu et al. 2017). The F_IS_ values, which are effectively zero for each collecting location (Table 1), indicate that inbreeding is limited within *E. larynx*, at least at the scale of a whole locale. Despite this low diversity, our calculated between‐colony pairwise F_ST_ values within each locale (Table S6A–D) appear elevated and are higher or comparable to population‐level comparisons in the above studies on other marine invertebrates, and comparable to microsatellite‐based F_ST_ values for distinct populations of the hydrozoan *Macrorhynchia phoenicea* (Postaire et al. 2017). This suggests that different colonies of *E. larynx* have distinct allele frequencies for the few sites that are polymorphic in a given collecting location.

**Table 1:** Per‐collecting‐location genetic diversity statistics

Location	# Polyps	Colonies	# SNPs	% Polymorphic sites	Π	F_IS_
*ME.2*	39	7	164	0.7373	0.002	−0.0005
*IRE.2*	14	4	32	0.2794	0.0008	0
*IRE.1*	14	3	100	0.3050	0.001	0.0001
*ME.1*	55	12	58	0.7764	0.0012	−0.0001

### GENOMIC MEASURES OF ALLELE‐SHARING WITHIN AND BETWEEN COLONIES

**Figure 4:**
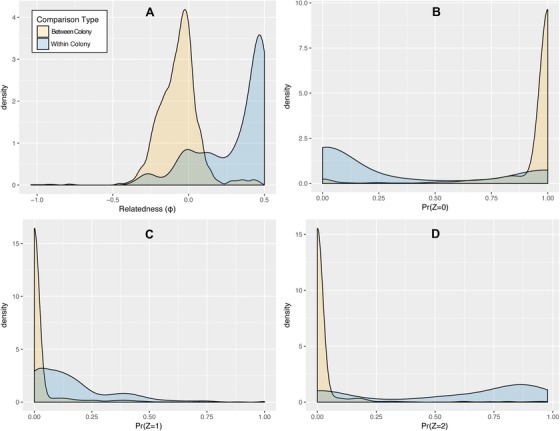
Distributions of values for several measures of relatedness for within‐ and between‐colony comparisons between individuals. Panel (A) displays values of the relatedness coefficient (equivalent to one half of Hamilton's *r*) as calculated using the software package KING. Higher values for the coefficient indicate a higher degree of allele sharing between individuals in a comparison, ranging from 0.5 for identical clones and 0 (or negative values treated as 0) for unrelated polyps. Panels (B–D) depict probabilities of different levels of identity by descent, as calculated using the software package PLINK. Pr(*Z* = 0) is the probability that individuals in a given comparison will be identical at a randomly selected SNP at no alleles, Pr(*Z* = 1) is the probability that individuals will be identical at one allele, and Pr(*Z* = 2) is the probability that a randomly selected SNP will be identical at both alleles.

For both within‐ and between‐colony comparisons between polyps, SNP‐based measures of relatedness, as estimated through the probability of allele‐sharing between individuals at many independent sites across their genome, are summarized in Figure 4, and full results for each pairwise comparison for each analysis are available in Table S7. Considering relatedness (**φ**) as calculated using the KING‐robust algorithm (Fig. 4A), the degree to which polyps share alleles is significantly higher in within‐colony comparisons (mean within = 0.264, mean between = –0.067, *t* = 33.174, df = 569.38, *P*‐value < 2.2 × 10^–6^). For comparison, for a parent‐offspring or full‐sib relationship, **φ** is expected to be 0.25, and ranges from 0 (negative values are treated as 0) for unrelated individuals and 0.5 for monozygotic twins or, in our case, identical clones (Manichaikul et al. 2010). This comparison suggests that, on average, polyps in a given colony are approximately as related as parent‐offspring pairs or siblings. However, within most colonies there are also pairwise comparisons between polyps that have **φ** of roughly 0, indicating that there are in fact fused polyps in these colonies that have no familial relationship whatsoever to the other polyps in the colony.

Calculations of identity‐by‐descent (IBD) probabilities using PLINK (Purcell et al. 2007) confirms these finding (Fig. 4B–D). Notably, the distributions of these measures for between‐ and within‐colony comparisons between polyps had very different shapes but overlapped in their ranges. For example, for between‐colony comparisons, the probability that a random locus is not IBD at either allele for a pair of polyps (i.e., Pr(Z = 0)) is very close to 1.0, with a mean Pr(Z = 0) of 0.945. For within‐colony comparisons, however, the distribution for IBD probabilities are much more uniform and have slight increases in density close to 0 and 1.0 for both Pr(Z = 0) and Pr(Z = 2) (the probabilities that the number of alleles that are IBD at a random locus for a pair of polyps is 0 or 2, respectively; Fig. 4B and D), further suggesting the presence of at least two classes of relatedness within colonies.

Given these measures of relatedness in a given colony, it appears that on average, polyps within the same colony are more related than those from different colonies and may therefore encounter less opportunity for genetic conflict due to allelic mismatches at loci associated with genetic conflict. However, another distinct class of less‐closely related polyps also exist within a given colony, with similar estimates of relatedness as compared to between‐colony comparisons, and which tend to share no alleles at polymorphic sites. This indicates that there is still potential for genetic conflict between some polyps in a given colony.

## DISCUSSION

### GENETIC COMPOSITION OF AND LEVELS OF RELATEDNESS WITHIN ECTOPLEURA LARYNX COLONIES

Our results demonstrate that colonies of *E. larynx* are genetically chimeric, containing multiple distinct genotypes that fall into potentially three distinct classes of genetic relationships, resulting in groups of polyps that likely represent clone‐mates, offspring/siblings, as well as polyps with nonfamilial relationships. The different genetic relationships within a colony can be explained by the life history of *E. larynx*. Clones can arise early in development when a new polyp will undergo an initial round of determinate budding, resulting in four to six polyps (Pyefinch and Downing 1949; Petersen 1990). In addition, although the adult colony does not bud asexually, *E. larynx* polyps have remarkable regenerative capabilities (Tardent 1963) and new polyps can form via regeneration in response to injury (P. Cartwright, pers. obs.). This initial asexual growth and regeneration response likely explains the persistence of clonal genotypes in a given colony, but it appears that *E. larynx* colonies achieve increased size from polyp fusion, including fusion of polyps not closely related to the main colony genotype. Colonies with genetically distinct polyps are thus produced through the fusion of juvenile polyps either brooded from the mother or from, unexpectedly, unrelated neighboring colonies.

We also determined whether or not polyps within colonies have a greater probability of identity by descent (IBD) than polyps in different colonies. We found that levels of relatedness were on average higher within colonies than between, but that there were many examples of polyps present in the same colonies that did not share either allele at the few polymorphic sites recovered and therefore may differ at relevant “conflict” loci.

The finding that polyps are generally more related within a colony than between colonies, but that there are some less‐related polyps in a colony, suggests that *E. larynx* may possess a mechanism of conflict mediation that homogenizes chimeric polyps, such as somatic‐cell takeover (Buss 1982; Michod 1982). However, the life history of *E. larynx*, particularly the budding and wound repair, can also explain this level of genetic relatedness as they ensure that a subset of polyps in a colony will be clonal. Additionally, juvenile polyps are limited in dispersal and thus frequently, but not always, settle on parental colonies, resulting in a colony consisting of polyps that are closely related. This is similar to results from the seaweed *Chondrus chrispus* where limited dispersal of gametes results in levels of relatedness between males siring offspring with the same female which are higher than background relatedness (Krueger‐Hadfield et al. 2015).

Our result that multiple genotypes are commonly found in colonies of *E. larynx* stands in contrast with previously reported examples of genetic heterogeneity among colonial organisms. Reported genetic mosaicism in some anthozoan and hydrozoan corals are represented by one or a few genetic changes likely generated by somatic mutations (Puill‐Stephan et al. 2009; Schweinsberg et al. 2015, 2016, 2017), and not multiple distinct genotypes as reported here (Table S4). The level of chimerism discovered in *E. larynx* colonies approaches that of some red seaweeds, that are known to derive ecological benefits from chimerism and also appear able to limit the level of chimerism in certain tissues, perhaps reducing the burden of genetic conflict (González and Santelices 2017; Santelices et al. 2016).

### LOW GENETIC DIVERSITY AND SELF/NONSELF RECOGNITION

Genetic chimerism is predicted to be much less common than mosaicism (intraorganismal diversity due to somatic mutation) due to the wider genetic distances involved and the potential involvement of the immune system in preventing wholesale fusion of organisms from occurring (Santelices 2004). The prevalence of genetic chimerism amongst *E. larynx* colonies, however, raises the question of why self/nonself recognition mechanisms appear not to be operating. One notable result of our work is the discovery that *E. larynx* has low population‐level diversity (Table 1), even when compared to other cnidarians and invertebrates sampled at similar spatial scales (see Results).

Given this finding, it is possible that polyps of *E. larynx* in a local mating population are genetically similar enough that potential germline conflict is mitigated. Many experimental fusion studies of colonial animals such as the tunicate *B. schlosseri* and the hydrozoan *Hydractinia symbiologicarpus* (Cadavid et al. 2004; Lakkis et al. 2008; Rosengarten and Nicotra 2011; Taketa and De Tomaso 2015) and of certain reef‐building corals (Puill‐Stephan et al. 2009) show that level of relatedness is directly correlated with the capacity with which two genotypes will fuse with one another to form a chimera. In the hydrozoan *H. symbiolongicarpus*, shared allorecognition alleles largely explain whether colonies are able to form persistent chimeras (Cadavid et al. 2004). If all polyps of *E. larynx* in an area are genetically similar to one another, selection for mechanisms to keep colonies homogenous may be greatly reduced compared to selection for large colony size. Thus, it is possible that *E. larynx* colonies cannot differentiate between self and nonself due to low genetic diversity.

Our measures of genome‐wide relatedness (see “Genetic Composition” section above), can serve as a proxy for the potential for genetic conflict at such loci, in the absence of knowledge in *E. larynx* about specific “conflict” loci such as those involved in allorecognition, and suggest the presence of a subset of polyps which in theory could differ at “conflict” loci within the same colony. Further work to identify and characterize actual allorecognition genes in the genome of *E. larynx* will shed light on whether or not more distantly related polyps in a given colony actually vary at sites important for self/nonself recognition.

One possible explanation for the low genetic diversity in populations of *E. larynx* is a climatically mediated genetic bottleneck, potentially at either geological time scales due to glaciation (Maggs et al. 2008), or at seasonal scales, due to harsh winter conditions causing mortality (Drolet et al. 2013). *E. larynx* is most abundant in late summer (Guenther et al. 2009) although it is unclear if this is entirely due to new colonies or recovery from a winter dormancy (Calder 1990).

### THE POTENTIAL FOR GENETIC CONFLICT WITHIN COLONIES OF ECTOPLEURA LARYNX

Given that *E. larynx* colonies appear to be made up of a set of relationships including the fusion of distinct genotypes, there is the potential for germline conflict. Within a colony, the multiple distinct genetic lineages may all be competing for the opportunity to be represented in the gametes. Given that hydrozoans continually produce new germline cells from a population of multi/totipotent stem cells (Müller et al. 2004), it is possible that any of the fused polyps may have access to gamete production in *E. larynx*. Barfield et al. (2016) found that colony‐specific somatic mutations in the coral *Orbicella faveolata* were not transferable to gametes, whereas Schweinsberg et al. (2014) demonstrated that more than one genotype from a colony of the coral *Acropora hyacinthus* was able to reproduce. Polyps in an *E. larynx* colony are almost always of the same sex, thus suggesting that a single germline is functioning. However, the relative contributions of environmental and genetic factors driving sex determination in hydrozoans is unclear (reviewed in Siebert and Juliano 2017). Future studies characterizing parental and offspring genotypes in *E. larynx* colonies are needed to definitively determine if germline chimerism or germline parasitism exists within an *E. larynx* colony.

## Conclusions

Past studies of the presence of polymorphism in cnidarian colonies have largely used a selection of mitochondrial and microsatellite markers, making this study among the first to capture genome‐scale information about intracolonial divergence and diversity. Our work reveals that colonies of *E. larynx* are genetically chimeric and contain multiple types of within‐colony genetic relationships, namely clones and familial relationships and, surprisingly, fusion of unrelated polyps from the local population with a low degree of allele‐sharing with the rest of the colony. This is consistent with a colony formation mechanism that relies on fusion of sexually produced offspring from the surrounding population, and not just the fusion of recently released brooded juvenile polyps from the parent colony. Due to observation of frequent within‐colony polymorphism and chimerism in *E. larynx*, and the observation that there are multiple levels of genetic relatedness in a colony, it appears that there is potential for germline‐soma conflict, but that this might be mitigated by low genetic diversity in populations of *E. larynx* and by processes that cause polyps in colonies to be more closely related on average than those from different colonies. Taken together, all of these results are consistent with the interpretation that there are multiple biological processes, including initial asexual budding, regeneration, fusion, and possibly a potential genetic conflict resolution mechanism, all contributing to the reevolution of *E. larynx*’s large colony size from a solitary ancestor.

## DATA ACCESSIBILITY

Raw sequence files for each individual polyp included in this study are available on NCBI Genbank under accession numbers SAMN08122681‐SAMN08122802. Intermediate files will be made available on DataDryad and results of analyses of genome‐wide relatedness are included here as a supplemental file.

Associate Editor: S. Wright

## Supporting information


**Table S1**. Collection sites of *Ectopleura* colonies from the coast of Maine and Ireland.
**Table S2**. Summary of loci for each colony‐level data se.
**Table S3**. Description of data sets used in each analysis in this publication.
**Figure S1 (previous page)**. Genetic relationships within selected colonies of *E. larynx* not already included in Figure 2.
**Table S4**. Summary of major per‐colony results.
**Figure S2**. Comparison of read‐depth distributions between SNPs where polyps were alternative homozygotes vs. all other SNPs included in the analysis.
**Figure S3**. Histogram of the number of sites at which polyps differ in comparisons between colonies at a given collecting site (orange) or within a colony (teal).
**Figure S5**. Results of different methods for choosing a best k (number of clusters) and the inferred cluster membership for K=2 for the proportion of sites which differ in a within‐colony comparison, for all within‐colony comparisons.
**Table S5**. Number of clusters (K) for the for the proportion of sites which differ in a within‐colony comparison, for all within‐colony comparisons, as inferred by a selection of algorithms employed by the NbClust() R package.
**Table S6A**. Between‐colony pairwise Fst values for collecting site ME1.
**Table S6B**. Between‐colony pairwise Fst values for collecting site ME2.
**Table S6C**. Between‐colony pairwise Fst values for collecting site IRE1.
**Table S6D**. Between‐colony pairwise Fst values for collecting site IRE2.
**Table S7**. Complete pairwise calculations of genome‐wide relatedness between polyps in each colony of *E. larynx*.

Supporting Information

Supporting Information

Supporting Information
