# Supplementary material for: Nonclonal coloniality: Genetically chimeric colonies through fusion of sexually produced polyps in the hydrozoan Ectopleura larynx
Source: Evol Lett. 2018 Jul 11;2(4):442–55. doi: 10.1002/evl3.68 (PMC6121865; doi:10.1002/evl3.68)
Supplement: Supplementary file 1 — Table S1. Collection sites of Ectopleura colonies from the coast of Maine and Ireland. Table S2. Summary of loci for each colony‐level data se. Table S3. Description of data sets used in each analysis in this publication. Figure S1 (previous page). Genetic relationships within selected colonies of E. larynx not already included in Figure 2. Table S4. Summary of major per‐colony results. Figure S2. Comparison of read‐depth distributions between SNPs where polyps were alternative homozygotes vs. all other SNPs included in the analysis. Figure S3. Histogram of the number of sites at which polyps differ in comparisons between colonies at a given collecting site (orange) or within a colony (teal). Figure S5. Results of different methods for choosing a best k (number of clusters) and the inferred cluster membership for K=2 for the proportion of sites which differ in a within‐colony comparison, for all within‐colony comparisons. Table S5. Number of clusters (K) for the for the proportion of sites which differ in a within‐colony comparison, for all within‐colony comparisons, as inferred by a selection of algorithms employed by the NbClust() R package. Table S6A. Between‐colony pairwise Fst values for collecting site ME1. Table S6B. Between‐colony pairwise Fst values for collecting site ME2. Table S6C. Between‐colony pairwise Fst values for collecting site IRE1. Table S6D. Between‐colony pairwise Fst values for collecting site IRE2. Table S7. Complete pairwise calculations of genome‐wide relatedness between polyps in each colony of E. larynx. [file EVL3-2-442-s001.docx]

Supplemental Table 1. Collection sites of *Ectopleura* colonies from the coast of Maine and Ireland

| Abbreviation | Site Name | Town | Region | Latitude | Longitude |
| --- | --- | --- | --- | --- | --- |
| ME1 | Darling Marine Center | Walpole | Maine, USA | 43.94277778 | -69.56888889 |
| ME2 | Downeast Institute | Beal's Island | Maine, USA | 44.47888889 | -67.59666667 |
| IRE1 | University Marine Lab | Pontaferry | Northern Ireland | 54.380705 | -5.550311 |
| IRE2 | Sketrick Pontoon | Sketrick Island | Northern Ireland | 54.48916 | -5.646843 |

**Supplemental Table 2. Summary of loci for each colony-level data set**

| ***Collecting*** | ***Colony*** | ***Sex*** | ***Polyps*** | ***Loci*** | ***RD***^†^ | | |
| --- | --- | --- | --- | --- | --- | --- | --- |
| ***Site*** |  |  |  |  |  |  |  |
| **Maine 1** | ME1.1 | M | 5 | 772 | 276.3 | | |
|  |  |  |  |  |  | | |
|  | ME1.2 | M | 5 | 439 | 267.0 | | |
|  | ME1.3 | M | 5 | 430 | 279.5 | | |
|  |  |  |  |  |  | | |
|  | ME1.4 | M | 6 | 143 | 240.5 | | |
|  | ME1.5 | F | 7 | 287 | 480.5 | | |
|  |  |  |  |  |  | | |
|  | ME1.6 | F | 7 | 183 | 292.4 | | |
|  | ME1.7 | F | 8 | 336 | 291.3 | | |
|  |  |  |  |  |  | | |
|  | ME1.8 | F | 6 | 167 | 291.4 | | |
|  | ME1.9 | F | 5 | 152 | 207.7 | | |
|  | ME1.10 | M | 5 | 300 | 89.3 | | |
| ***N. Ireland 1*** | IRE1.1 | F | 4 | 270 | 88.5 | | |
|  | IRE1.2 | F | 6 | 244 | 112.7 | | |
| ***Maine 2*** | ME2.1 | M | 7 | 665 | 305.5 | | |
|  | ME2.2 | M | 6 | 607 | 214.3 | | |
|  | ME2.3 | F | 8 | 435 | 277.0 | | |
|  |  |  |  |  |  | | |
|  | ME2.4 | F | 5 | 553 | 190.7 | | |
|  | ME2.5 | H | 5 | 639 | 234.9 | | |
| ***N. Ireland 1*** | IRE2.2 | F | 5 | 132 | 266.3 | | |
|  | IRE2.3 | M | 5 | 326 | 94.4 | | |

|  | † Average read depth per polymorphic site/per individual at site. H=Hermaphroditic colony with |
| --- | --- |

both male- and female polyps.

Supplemental Table 3. Description of data sets used in each analysis in this publication

| **Analysis** | **Stacks Populations module filtering for loci** | **Number of Individuals** | **Number of Loci** |
| --- | --- | --- | --- |
| **Colony-level analyses (Rclone, per-locus study)** | Prepared per colony, all polyps in a given colony, m=8 | Varies by colony, see Table 1 | Varies by colony, see Table 1 |
| **Collecting Site Diversity Statistics (Table 2 and Figure 4)** | Prepared per collecting site., r=1.0, m=6 | See Table S2 | See Table 2 |
| **Collecting-site level pairwise F_ST_** | Prepared per collecting site., r=1.0, m=6 | See Table S2 | Varies by pairwise comparison |
| **PLINK and KING analyses of genetic relatedness** | Prepared per collecting site, r=.75, m=4, p=.8, --write_single_snp | See Table S2 | ME1=954, ME2=1025, IRE1=328, IRE2=364 |


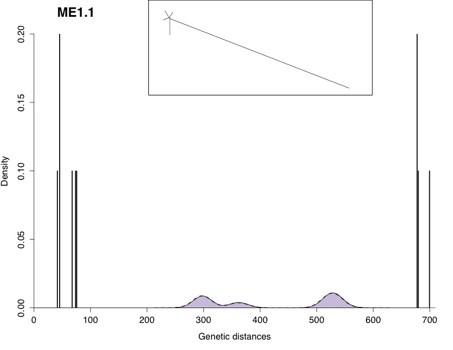

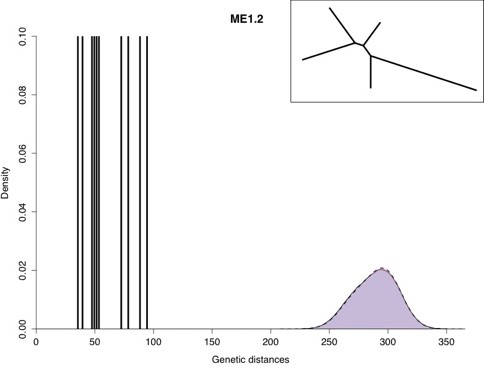

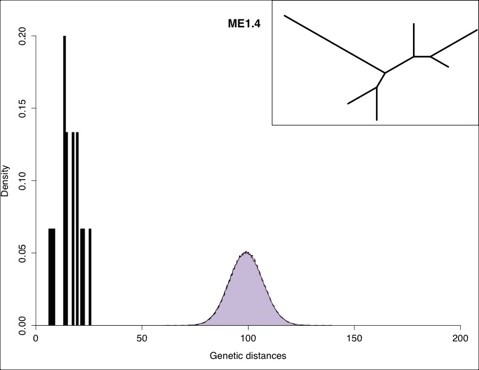

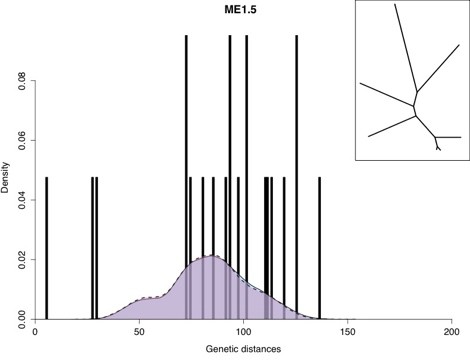

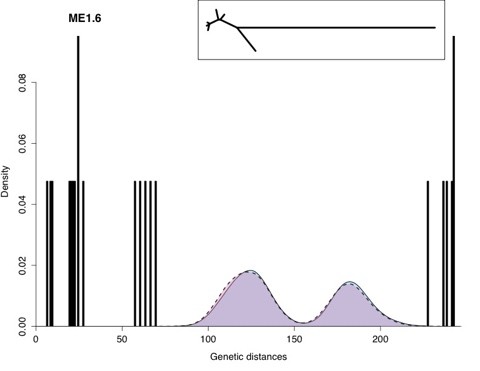

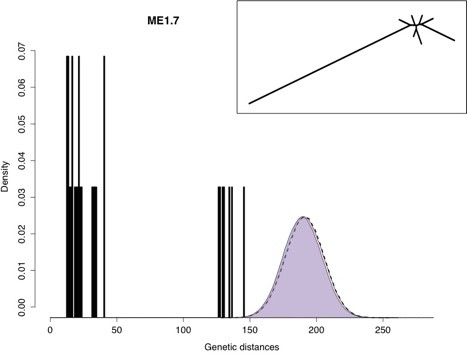

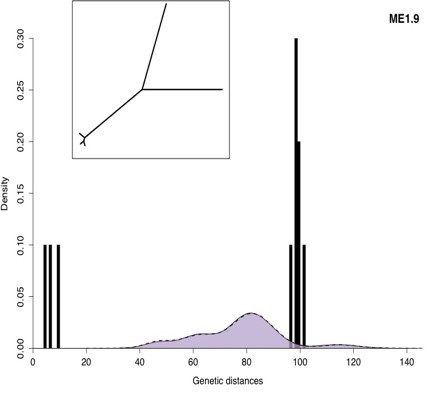

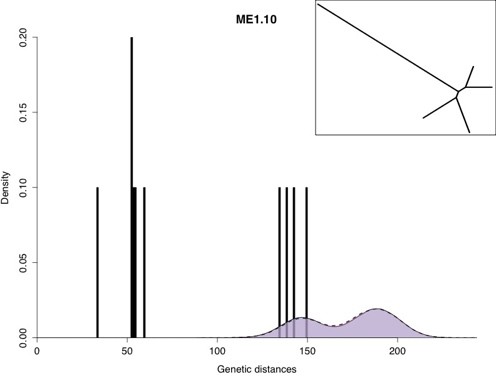

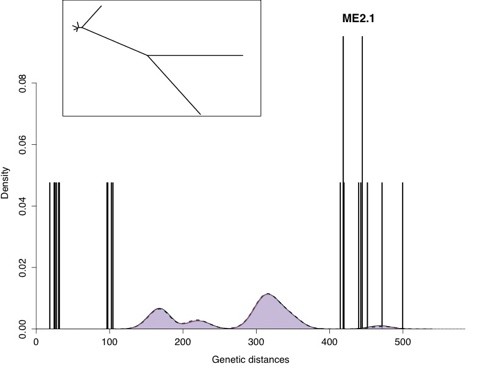

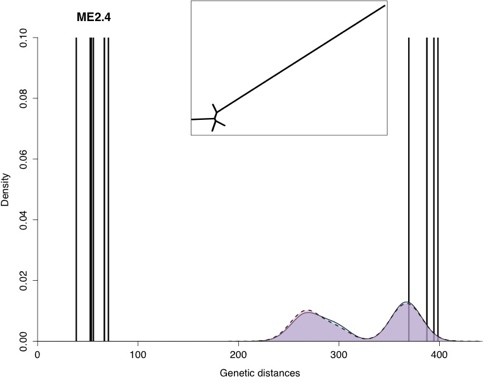

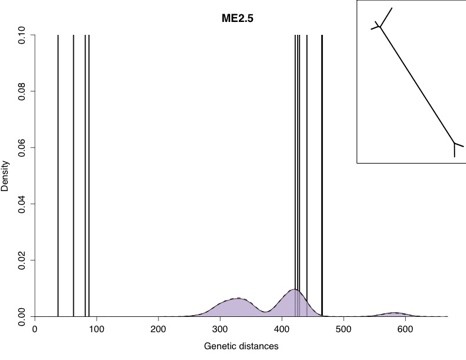

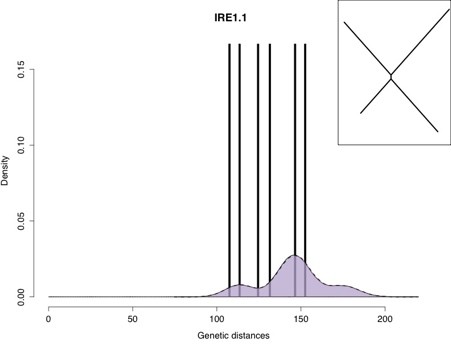

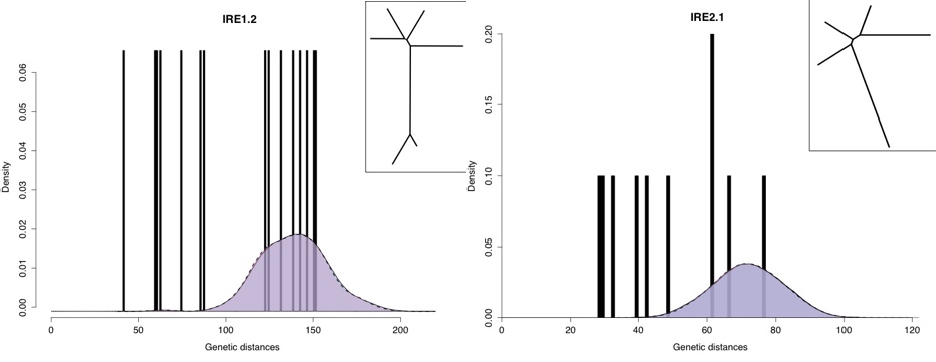

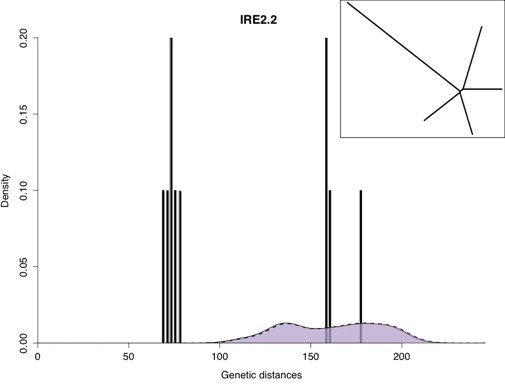


**Supplemental Figure 1 (previous page). Genetic relationships within selected colonies of *E. larynx* not already included in Figure 2*.*** Actual genetic distances (black bars) between polyps of *E. larynx* in every possible pairwise comparison in a colony, and those predicted under simulations of sex with selfing (pink) and without (blue) (purple=overlap of distribution). Insets are neighbor-joining trees with each branch representing a polyp within the colony. Colony designations are as in Tables 1 and S2.

**Supplemental Table 4.** Summary of major per-colony results

COLONY ID

TYPES OF RELATIONSHIPS

EVIDENCE OF GENE FLOW

RATIO OF UNIQUE GENOTYPES TO POLYPS SAMPLED

| **ME1.1** | I,III | Yes | 0.4 |
| --- | --- | --- | --- |
| **ME1.2** | I | No | 0.2 |
| **ME1.3** | I | No | 0.2 |
| **ME1.4** | I | No | 0.17 |
| **ME1.5** | I,II,III | Yes | 0.717 |
| **ME1.6** | I,II,III | Yes | .43 |
| **ME1.7** | I,II | Yes | 0.25 |
| **ME1.8** | I,II | Yes | 0.34 |
| **ME1.9** | I,II | Yes | 0.4 |
| **ME1.10** | I,II | Yes | 0.4 |
| **ME2.1** | I,II,III | Yes | 0.57 |
| **ME2.2** | I,III | Yes | 0.34 |
| **ME2.3** | I,II,III | Yes | 0.38 |
| **ME2.4** | I,II | Yes | 0.4 |
| **ME2.5** | I,II | Yes | 0.4 |
| **IRE1.1** | II | Yes | 1 |
| **IRE1.2** | I,II | Yes | 0.5 |
| **IRE2.1** | I,II | Yes | 0.4 |
| **IRE2.2** | I,II | Yes | 0.6 |


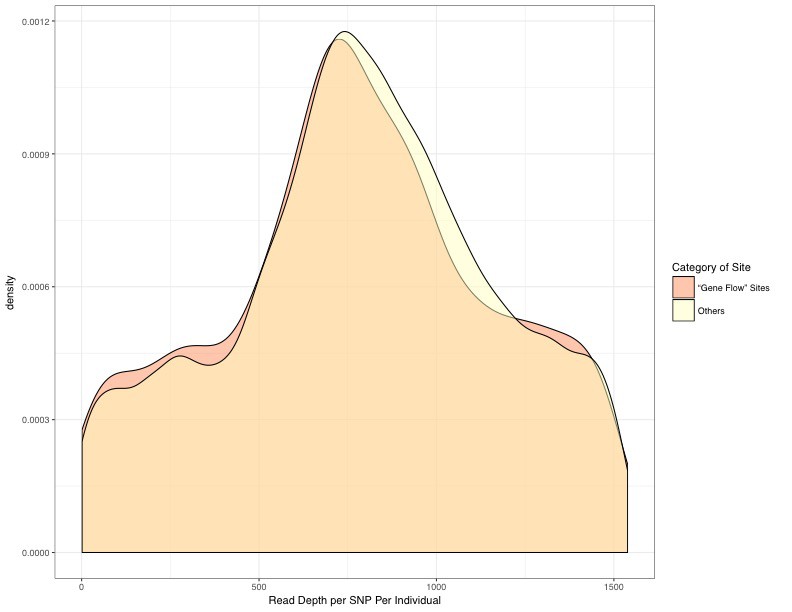


**Supplemental Figure 2**. **Comparison of read-depth distributions between SNPs where polyps were alternative homozygotes vs. all other SNPs included in the analysis.** SNPs were taken to be evidence of polyp fusion into a particular colony if at least one polyp in the colony differed from the clonal genotype at this SNP by more than one mutational/error step (that is, polyps shared no alleles at this SNP). SNPs were added to the data set for this figure in a per-colony basis. That is, the only read depths considered for the potential sites were those depths associated with that particular colony in which the pattern appeared at that site, and the sites in that colony that were not the alternate homozygote were included as the “others”.


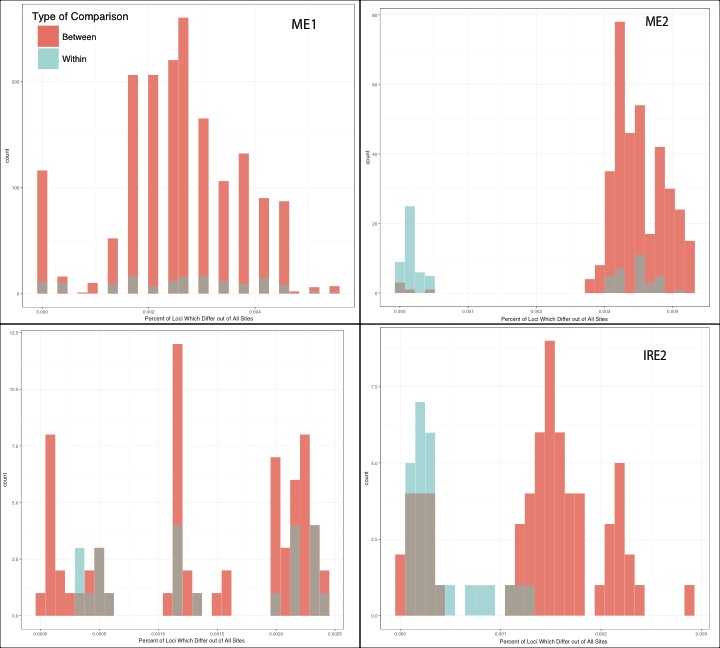


**Supplemental Figure 3. Histogram of the number of sites at which polyps differ in comparisons between** **colonies at a given collecting site (orange) or within a colony (teal).** Data sets are the same as for main Table 2.

**Supplemental Figure 5. Results of different methods for choosing a best k (number of clusters) and the inferred cluster membership for K=2 for the proportion of sites which differ in a within-colony comparison, for all within-colony comparisons.** Data set used is the same as for colony level analysis (see Supplemental Table 3). Panels depict graphical representations of several methods of selecting the minimal number of clusters needed to explain most of the variation in our data set.

**Supplemental Table 5: Number of clusters (K) for the for the proportion of sites which differ in a within-colony comparison, for all within-colony comparisons, as inferred by a selection of algorithms employed by the NbClust() R package.**

| **method** | **K inferred** |
| --- | --- |
| kl | 8 |
| ch | 0 |
| hartigan | 4 |
| mcclain | 2 |
| gamma | 2 |
| gplus | 2 |
| tau | 4 |
| dunn | 2 |
| sdindex | 3 |
| sdbw | 3 |
| cindex | 9 |
| silhouette | 2 |
| ball | 3 |
| ptbiserial | 2 |
| gap | 2 |
| frey | 2 |

**Supplemental Table 6A. Between-colony pairwise Fst values for collecting site ME1**

|  | **ME1.1** | **ME1.9** | **ME1.10** | **ME1.2** | **ME1.3** | **ME1.4** | **ME1.5** | **ME1.6** | **ME1.7** | **ME1.8** |
| --- | --- | --- | --- | --- | --- | --- | --- | --- | --- | --- |
| **ME1.1** |  | 0.0945748 | 0.0495127 | 0.183793 | 0.170983 | 0.156707 | 0.0518077 | 0.090722 | 0.190072 | 0.156566 |
| **ME1.9** |  |  | 0.0936853 | 0.196223 | 0.196624 | 0.152959 | 0.0850591 | 0.153255 | 0.165496 | 0.191157 |
| **ME1.10** |  |  |  | 0.165463 | 0.1533 | 0.14201 | 0.066391 | 0.116256 | 0.170984 | 0.142545 |
| **ME1.2** |  |  |  |  | 0.277778 | 0.302979 | 0.119037 | 0.213865 | 0.288889 | 0.286382 |
| **ME1.3** |  |  |  |  |  | 0.280773 | 0.0908441 | 0.195026 | 0.255411 | 0.233626 |
| **ME1.4** |  |  |  |  |  |  | 0.0899364 | 0.225502 | 0.249395 | 0.265887 |
| **ME1.5** |  |  |  |  |  |  |  | 0.0803301 | 0.0902062 | 0.0775534 |
| **ME1.6** |  |  |  |  |  |  |  |  | 0.261914 | 0.200205 |
| **ME1.7** |  |  |  |  |  |  |  |  |  | 0.208586 |

**Supplemental Table 6B. Between-colony pairwise Fst values for collecting site ME2**

|  | **ME2.1** | **ME2.2** | **ME2.3** | **ME2.4** | **ME2.5** |
| --- | --- | --- | --- | --- | --- |
| **ME2.5** |  | 0.142421 | 0.0870675 | 0.146802 | 0.115565 |
| **ME2.6** |  |  | 0.148295 | 0.176256 | 0.18661 |
| **ME2.7** |  |  |  | 0.120654 | 0.129049 |
| **ME2.8** |  |  |  |  | 0.123994 |

**Supplemental Table 6C. Between-colony pairwise Fst values for collecting site IRE1**

|  | **IRE1.1** | **IRE1.2** |
| --- | --- | --- |
| **IRE1.1** |  | 0.256271 |

**Supplemental Table 6D. Between-colony pairwise Fst values for collecting site IRE2**

|  | **IRE2.1** | **IRE2.2** |
| --- | --- | --- |
| **IRE2.1** |  | 0.273354 |
