# Supplementary material for: Nonclonal coloniality: Genetically chimeric colonies through fusion of sexually produced polyps in the hydrozoan Ectopleura larynx
Source: Evol Lett. 2018 Jul 11;2(4):442–55. doi: 10.1002/evl3.68 (PMC6121865; doi:10.1002/evl3.68)
Supplement: Supplementary file 2 — Supporting Information [file EVL3-2-442-s002.docx]

**Details of Rad-Seq Library Preparation:**

Use of the NdeI restriction enzyme (NEB Biolabs, Ipswich, MA,USA) for DNA digestion, a total of 14 rounds of PCR on the pooled products, and the addition of a 10% PhiX spike-in to increase library complexity for sequencing. To allow for demultiplexing of sequences from different samples, each sample was ligated to one of 48 unique barcode adaptors, and each such set of 48 samples was pooled independently. Each of these sublibraries was then combined with different Illumina indices during the PCR step of the library preparation (Monnahan et al. 2015).

**Details about calculating population-wide estimates of diversity:**

For the data sets used to calculate collecting-location-level genetic diversity statistics, every individual available at a given site was incorporated into the analyses, even if that polyp’s colony was not included in colony-level analyses. Minimum read depth was decreased to six (-m 6) and coverage across all polyps in the whole location (-r 1.0) was required for a SNP to be retained. Genetic diversity statistics reported in Table 3 were taken directly from the output of the Stacks “populations” module for each relevant collecting location. Additionally, these location-level data sets were used to calculate pairwise between-colony F_ST_ values also using the Stacks “populations” module. For a brief summary of data sets and filtering parameters for each analysis conducted in this publication, see Supplemental Table 3.

**Creating RClone Figures:**

Specifically, Structure-formatted output files from Stacks for each colony were processed into genind files using Adegenet R package v2.0.1 (Jombart 2008; Jombart and Ahmed 2011). Using the genet_dist() function of Rclone all possible pairwise allelic distances between polyps in a colony were calculated, and then, using the actual within-colony data, the distribution of those distances with and without selfing (occurring at a rate determined by the number of clonal replicates in the sample) were simulated using the genet_dist_sim() function with 1000 simulations each for each colony separately. Density plots of the actual data and simulated distributions were made using the base R graphics library. Neighbor-joining trees using the distance matrices created by RClone were created for each colony using the nj() function from the ape R package v4.1 (Paradis et al. 2004).

**Description of tracing patterns of allelic segregation within a colony:**

We developed custom R scripts that first evaluated whether polymorphisms between putative clones in a colony (polyps with smaller genetic distances between them than those generated by RClone assuming sexual reproduction) were shared or not between different pairwise comparisons between clones. For example, random polymorphisms in each comparison not shared by other comparisons would be indicative of random sequencing error. Next the most common genotype at each SNP was taken as the “standard clonal genotype” for each colony to avoid the effects of sequencing error when comparing with other genotypes found within the colony. After the clonal genotype was established for a given colony, SNPs that differed between the “standard clonal genotype” were classified as either re-assortment of the two alleles or as having non-familial alleles indicative of other error/mutation/or gene flow from another colony. The SNPs with non-familial alleles were further divided into whether or not they differed by one or both alleles from the parental genotype.

**LITERATURE CITED**

Jombart, T. 2008. adegenet: a R package for the multivariate analysis of genetic markers. Bioinformatics 24:1403-1405.

Jombart, T. and I. Ahmed. 2011. adegenet 1.3-1: new tools for the analysis of genome-wide SNP data. Bioinformatics 27:3070-3071.

Knaus, B. J. and N. J. Grünwald. 2017. vcfr: a package to manipulate and visualize variant call format data in R. Mol Ecol Res 17:44-53.

Paradis, E., J. Claude, and K. Strimmer. 2004. APE: Analyses of Phylogenetics and Evolution in R language. Bioinformatics 20:289-290.
